# Transcriptome analysis reveals the sex-switching mechanism of juvenile hermaphroditism in silver pomfret (*Pampus argenteus*)

**DOI:** 10.1186/s13293-025-00736-1

**Published:** 2025-07-14

**Authors:** Yaya Li, Jiabao Hu, Chongyang Wang, Man Zhang, Youyi Zhang, Yuanbo Li, Mengke Tang, Chunlai Qin, Zukang Feng, Shanliang Xu, Xiaojun Yan, Xubo Wang, Haimin Chen, Yajun Wang

**Affiliations:** 1 College of Marine Sciences, Ningbo University, Ningbo, China; 2 Key Laboratory of Applied Marine Biotechnology, Ningbo University, Ministry of Education, Ningbo, China; 3 Key Laboratory of Marine Biotechnology of Zhejiang Province, Ningbo University, Ningbo, China; 4 Ningbo Ocean Research Institute, Ningbo, China

**Keywords:** Sex differentiation, Juvenile hermaphroditism, Apoptosis, Sex hormone, *Pampus argenteus*

## Abstract

**Background:**

Our previous study on silver pomfret (*Pampus argenteus*) demonstrated that all gonads initially develop into ovaries by 60 days post-hatch (dph). Between 80 and 120 dph, some oocytes undergo apoptosis, resulting in the development of testes and a transient hermaphroditic stage. This observation indicates a complex molecular mechanism underlying sex differentiation in this species.

**Methods:**

Gonadal samples were collected at 90 dph, 120 dph, and 150 dph, with sex identification performed by HE staining and transcriptome sequencing. Morphological traits, including body length and weight, were measured to evaluate sexual size dimorphism. Candidate genes related to with sex differentiation were identified through differential gene expression analysis and feature selection methods, followed by gene set enrichment analysis to identify potential molecular pathways. Heatmaps were generated to visualize gene expression patterns across developmental stages and samples. Sex hormones concentrations were measured using commercial assay kits to assess their role in gonadal differentiation. RT-qPCR validated the sequencing results, while immunofluorescence (IF) examined the expression of related genes in testes and ovaries.

**Results:**

Histological and transcriptomic analyses identified the period between 90 and 120 dph as critical for sex differentiation in silver pomfret. At 90 dph, apoptotic signals trigger the apoptosis of early-stage oocytes. During this period, both testis-preferential and ovary-preferential genes exhibit high expression, leading to spermatogonia differentiation and the emergence of a juvenile hermaphroditic stage. The study established that androgens 11-KT and estrogen E2 regulate sex differentiation through modulation of related gene expression, with 11-KT serving a crucial role. This process involved significant enrichment of the steroid hormone biosynthesis pathway and the metabolism of xenobiotics by cytochrome P450 in the testes. Ovarian development is characterized by fatty acid metabolism and PPAR signaling pathway activation, along with energy metabolism pathways including the citrate cycle (TCA cycle) and cell degradation processes, such as lysosome activity and ubiquitin-mediated proteolysis, suggesting that ovarian development encompasses lipid accumulation and follicular selection.

**Conclusions:**

This investigation illuminates the molecular processes underlying this distinctive pattern of gonadal differentiation, providing novel insights into sex differentiation in fish exhibiting a juvenile hermaphroditic stage.

**Graphical Abstract:**

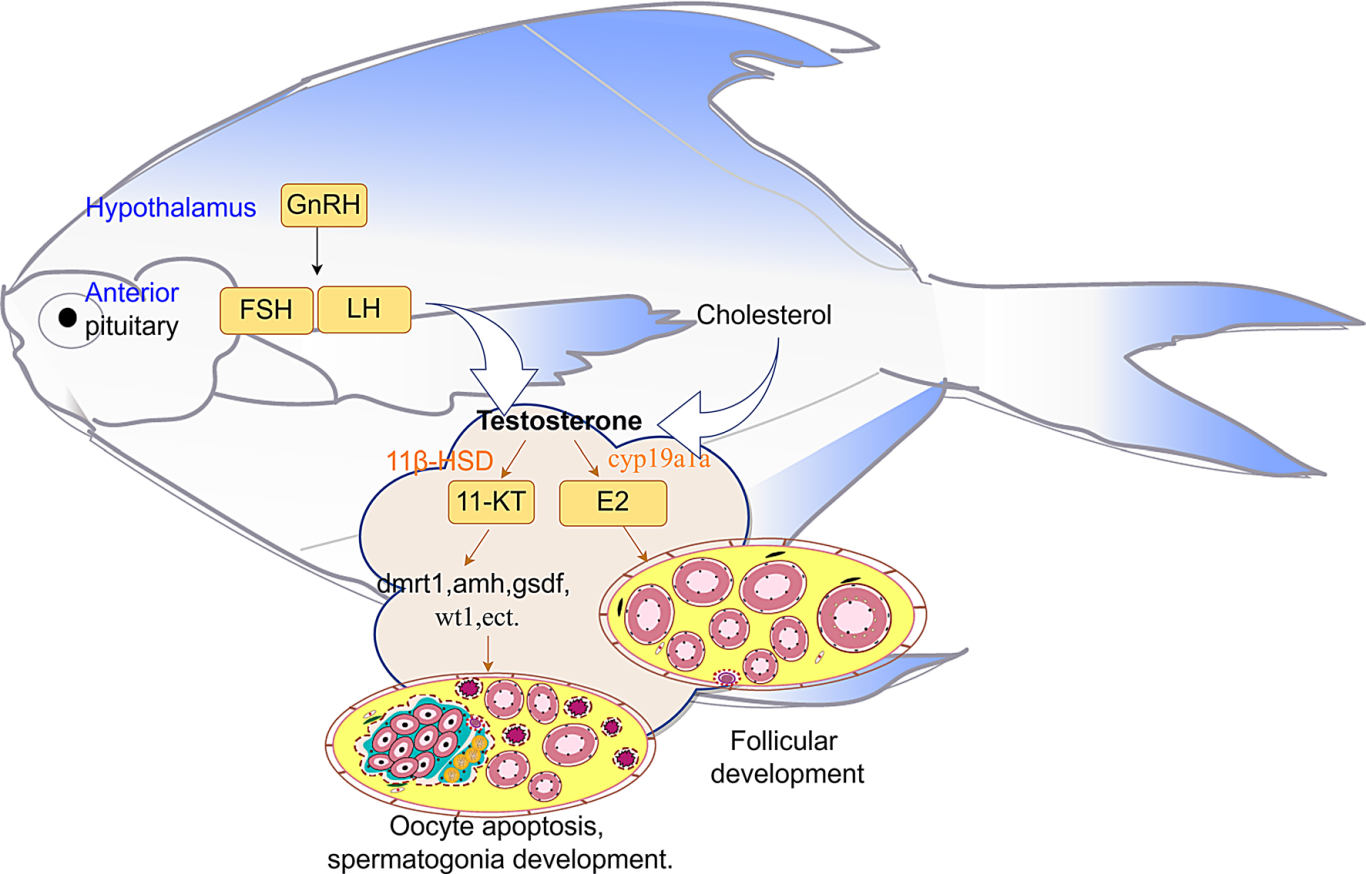

**Supplementary Information:**

The online version contains supplementary material available at 10.1186/s13293-025-00736-1.

## Plain Language summary

Juvenile hermaphroditism represents an uncommon sex differentiation pattern, characterized by testis differentiation following oocyte apoptosis and a juvenile hermaphroditic phase. This study reveals that metabolic changes and increased expression of testis differentiation-related genes (*dmrt1*, *wt1*, *gsdf*, ect) contribute significantly to oocyte apoptosis and juvenile hermaphroditism in 90 dph male fish. The research demonstrates that sex hormones 11-KT and E2 influence gonadal differentiation through regulation of sex differentiation-related gene expression, with 11-KT serving a central role. The simultaneous elevated expression of 11-KT and E2 potentially explains the sustained juvenile hermaphroditic phase in certain fish. Conversely, ovarian development encompasses energy accumulation and oocyte natural selection. This investigation provides comprehensive understanding of sex-switching mechanisms in juvenile hermaphroditic fish, establishing groundwork for future research into this distinctive gonadal differentiation pattern.

**Supplementary Information:**

The online version contains supplementary material available at 10.1186/s13293-025-00736-1.

## Highlights

Histological evidence of sexual differentiation was examined in silver pomfret.

Metabolic changes and the expression of apoptotic signals are the primary causes of early-stage oocyte apoptosis.

The simultaneous high expression of testis and ovary differentiation-related genes led to the formation of juvenile hermaphroditism.

Sex hormones play a crucial role in regulating the sex differentiation of silver pomfret.

**Supplementary Information:**

The online version contains supplementary material available at 10.1186/s13293-025-00736-1.

## Introduction

Sexual dimorphism represents a prominent characteristic of aquatic species, with growth, size, and immune responses frequently correlating with sexual dimorphism across numerous fish [1, 2]. Sexual size dimorphism occurs commonly in fish, and monosex breeding effectively enhances the economic value of species exhibiting this trait [3, 4]. Fish exhibit three main sex determination mechanisms: genotypic sex determination (GSD), environmental sex determination (ESD), and their combination (GSD + ESD). In GSD species, sex determination primarily occurs through sex-determining genes on sex chromosomes. Monosex populations can be successfully produced through genetic editing techniques [5], gynogenesis [3], and crossbreeding [6]. For ESD species, where environmental factors significantly influence sex determination, achieving stable monosex breeding remains technically challenge.

Silver pomfret (*Pampus argenteus*), distributed throughout South Asia, Southeast Asia, and the Middle East, represents an economically important fish in these regions. Since 2016, researchers at Ningbo University have achieved significant advances in silver pomfret breeding, enabling artificial cultivation [7]. During cultivation, silver pomfret demonstrates marked sexual size dimorphism, with females substantially larger than males [8]. Consequently, developing all-female breeding methods would enhance industry development. However, our research indicates that silver pomfret follows ESD, with sex differentiation influenced by nutritional factors [8], currently preventing monosex breeding achievement. Furthermore, silver pomfret exhibits a distinctive sex differentiation process, initially developing into ovarian-like structures. At 80 dph, certain fish undergo oocyte apoptosis. Between 90 and 120 dph, remaining oocytes coexist with developing spermatogonia, producing rare hermaphroditism [8]. Similar patterns appear in various fishes, including zebrafish [9], little yellow croaker [10], *Gymnocorymbus Ternetzi* [11] and European sea bass [12]. Thus, as an ESD fish demonstrating rare juvenile hermaphroditism during sex differentiation, silver pomfret provides valuable research material for understanding fish sex determination. Investigation of its sex differentiation mechanism advances breeding industry development and enhances understanding of fish hermaphroditism.

Through extensive research efforts, scientists have identified sex-determining genes in various GSD fish species. In 2002, researchers discovered the sex-determining gene DMY in *Oryzias latipes* [13]. Further investigations revealed additional sex-determining genes, including doublesex and mab-3 related transcription factor 1 (*dmrt1*) [14, 15], anti-Mullerian hormone (*amh*) [16, 17], gonadal somatic cell derived factor (*gsdf*^Y^) [18], the SD gene (*sd*^*Y*^) [19], the PDZ domain-containing gene (*pfpdz1*) [20], the Y-linked BMP1B receptor (*bmpr1bby*) [21], and growth/differentiation factor 6-B (*gdf6b*) [22]. These discoveries underscore the species-specific nature and complexity of sex determination mechanisms in fish. Notably, these sex-determining genes are found exclusively in strictly gonochoristic fish species. Hermaphroditic fish seem to lack heteromorphic chromosomes and major sex-determining genes. Studies on zebrafish [23], European seabass [24], and several cichlid species from Lake Malawi [25] demonstrate that polygenic sex determination (PGSD) is the primary mechanism governing sex differentiation in hermaphroditic fishes. In these species, genes affecting gonadal differentiation are distributed across the genome. Furthermore, multiple pathways, including the wnt signaling pathway [26], the steroid hormone synthesis signaling pathway [27], the TGF-beta signaling pathway [28], and various immune pathways [26], participate in the sex differentiation process. These observations indicate that sex differentiation in fish results from the coordinated regulation of multiple genes and signaling pathways.

While extensive research has examined the sex differentiation process in fish with a hermaphroditic stage and identified candidate sex-determining genes, the detailed switch processes in juvenile hermaphroditic fish require further investigation. This research initially identified the gonadal development stages of silver pomfret through histological sections. Subsequently, transcriptome analysis was performed to identify Kyoto Encyclopedia of Genes and Genomes (KEGG) pathways and differentially expressed genes (DEGs) associated with gonadal differentiation, thereby illuminating the underlying molecular mechanisms. Immunohistochemistry demonstrated differential expression of mitochondrial metabolism-related genes in early gonads. The study also examined differences in serum sex hormone levels between male and female fish during the critical period of gonadal differentiation. This investigation presents the first systematic analysis of juvenile hermaphroditism causes in silver pomfret, offering valuable insights for studies on other fish species in the juvenile hermaphroditic stage and establishing a foundation for silver pomfret industrialization.

## Materials and methods

### Silver pomfret gonad sampling

Healthy silver pomfrets were cultivated at Xiangshan Bay Aquatic Seedlings Co., Ltd. in Ningbo. The juvenile silver pomfrets were maintained indoors in 3000 L ponds with sand-filtered seawater (temperature, 25 ± 0.5 °C; pH, 8.2 ± 0.3; dissolved oxygen, 7.3 ± 0.05 mg/L; salinity, 25 ± 0.5‰) and received commercial feed (larve love, 6#, Japan) six times daily at 2–3% of their body weight. To comprehensively investigate the sex differentiation mechanism of silver pomfret, gonad samples were collected from 30 fish at 90, 120, and 150 dph. During sampling, silver pomfrets underwent anesthesia with MS222 (Sigma, Shanghai, China). Body length and weight were measurements recorded, and venous blood was collected. The fish were disinfected with 75% ethanol before gonad tissue extraction. Half of the gonadal tissue was preserved in 4% paraformaldehyde (Solarbio, Beijing, China) for subsequent histological analysis to determine gonadal developmental stages. The remaining halves were stored in liquid nitrogen for transcriptome sequencing and real-time PCR (RT‒qPCR) analysis based on gonadal developmental stages.

### Histological analysis

The gonad tissues fixed in 4% paraformaldehyde underwent dehydration in a gradient ethanol series (Sinopharm, Beijing, China), clearing in xylene (Sinopharm) and embedding in liquid paraffin. The samples were then sectioned into 5 μm-thick slices. After being stained with hematoxylin (Solarbio) and eosin (Servicebio, Wuhan, China), the sections were mounted with neutral resin (Servicebio). Observation was performed using a microscope (Nikon, Tokyo, Japan), and images were captured with a SPOT FLEX camera.

### Transcriptome sequencing and analysis

#### RNA extraction, cDNA library construction, and sequencing

The gonad tissues preserved in liquid nitrogen underwent RNA extraction using TRIzol^®^ reagent (Invitrogen, CA, USA). RNA quality and concentration were evaluated using 1% agarose gel electrophoresis and a NanoPhotometer^®^ spectrophotometer (IMPLEN, CA, USA). RNA integrity was thoroughly assessed using an Agilent 2100 bioanalyzer. Sequencing libraries were prepared using the NEBNext^®^ Ultra™ RNA Library Prep Kit. Initially, polyA-tailed mRNA was isolated using oligo(dT) magnetic beads. The isolated mRNA was randomly fragmented in fragmentation buffer containing divalent cations. The fragmented mRNA was used as a template to synthesize the first strand of cDNA using random oligonucleotide primers and M-MuLV reverse transcriptase. DNA polymerase I was then used to synthesize the second strand of cDNA. The purified double-stranded cDNA underwent end repair, A-tailing, and adaptor ligation. AMPure XP beads were utilized to select cDNA fragments of approximately 370–420 bp, followed by PCR amplification. The PCR products underwent further purification using AMPure XP beads to obtain the final library. Library quantification was performed using a Qubit 2.0 fluorometer, and insert size was verified using an Agilent 2100 bioanalyzer for quality assurance. Following successful quality control, the library proceeded to Illumina sequencing.

#### Transcriptome data processing and analysis

Raw data underwent base recognition using CASAVA and were converted into reads. Reads containing adapters, undetermined bases, or those with low quality (Q value ≤ 20) were filtered, out to obtain clean data. The clean data were analyzed to determine the Q20, Q30, and GC contents. The paired-end clean reads were aligned to the silver pomfret genome (https://www.ncbi.nlm.nih.gov/datasets/genome/GCA_036937835.1/) [29] using HISAT2 v2.0.5. StringTie v1.3.3b was employed for new gene prediction. FeatureCounts (https://subread.sourceforge.net) was utilized to enumerate reads mapped to each gene [30]. Gene expression abundances and variations were calculated and normalized to the number of transcripts per kilobase of exon per million mapped reads.

DEGs analysis was conducted using the DESeq2 R package (1.20.0), with genes showing minimal twofold difference in expression level|log2[foldchange]| ≥ 1 and *q*-value < 0.01 between any two groups identified as DEGs. Gene Ontology (GO) enrichment and KEGG pathway analyses were performed using OmicShare tools (https://www.omicshare.com/tools) to determine DEG functions. GO terms and KEGG pathways with corrected *p*-values < 0.05 were considered significantly enriched among the DEGs. An interaction network was constructed using R package weighted gene coexpression network analysis (WGCNA) following established procedures [31]. Gene modules and expression correlation coefficients were calculated and established. GO term and KEGG pathway enrichment analyses were conducted.

### Quantitative real-time PCR (RT–qPCR) validation and statistical analysis

Following transcriptomic screening, sex differentiation-associated DEGs were selected for RT‒qPCR validation. Primers were designed using Primer 5.0 software, with detailed primer information provided in Table S1. RNA samples were reverse transcribed to cDNA using the NovoScript^®^ Plus All-in-one 1st Strand cDNA Synthesis SuperMix (gDNA Purge) (Novoprotein, Suzhou, China) according to manufacturer’s protocols. Gene expression differences were analyzed using NovoStart^®^ Universal Fast SYBR qPCR SuperMix (Novoprotein) and a real-time thermal cycler (Kubo Tech Co., Ltd., Beijing, China). NormFinder software (v0.953) was employed to identify appropriate reference genes for accuracy, with *β-actin* [32] selected as the optimal reference gene. The 2^−ΔΔCT^ method (*n* = 3) was applied to calculate relative expression levels. Statistical analysis was performed using one-way ANOVA followed by Tukey’s multiple range test (GraphPad Prism, version 10.0).

### Immunofluorescence

The wax blocks were sectioned into 5 μm-thick slices, followed by dewaxing in xylene and a graded ethanol series in water. Antigen retrieval was conducted using EDTA antigen repair solution. After blocking with BSA for 30 min, the sections were incubated overnight at 4 °C with Cox6a (cytochrome c oxidase subunit 6 A) and Cox5b (cytochrome c oxidase subunit 5B) antibodies (Servicebio, rabbit anti-human, 1:300). Following PBS washing, the sections were incubated with a goat anti-rabbit secondary antibody (Servicebio, 1:200) at room temperature for 50 min. DAPI staining solution was applied, and the sections were incubated at room temperature in darkness for 10 min. A tissue autofluorescence quenching agent (Beyotime, Shanghai, China) was added, followed by rinsing with running water for 10 min. The sections were mounted with antifade mounting medium (Beyotime) and imaged using a fluorescence microscope (Nikon).

### Measurement of steroid hormones

Venous blood samples were collected from silver pomfret at 90, 120, and 150 dph. After incubation at 4 °C for 4 h, the samples underwent centrifugation at 3,000 rpm to isolate the serum, which was stored at -80 °C. Sex determination was based on histological analysis. Serum levels of 17β-estradiol (E2), 11-keto-testosterone (11-KT), and testosterone (T) were measured using the Fish Elisa Kit (Nanjing Jiancheng Bioengineering Institute, Nanjing, China) according to the manufacturer’s protocols (*n* = 6).

### Statistical analysis

The data are presented as the means ± standard deviations (SDs). Data analysis was conducted using Prism, version 10.0 (GraphPad Software, Inc., La Jolla, CA, USA). Independent sample t-tests were used to evaluate differences between two groups, and one-way ANOVA was employed for comparisons among multiple groups for normally distributed data with homogeneous variance. For nonnormally distributed data, the Mann‒Whitney test was utilized to analyze differences between two groups. Statistical significance is indicated as *P ≤ 0.05, **P ≤ 0.01, ***P ≤ 0.001, and ****P ≤ 0.0001.

## Results

### Histological observation of gonadal differentiation in silver pomfret and sexual dimorphism in size

**Fig. 1:**
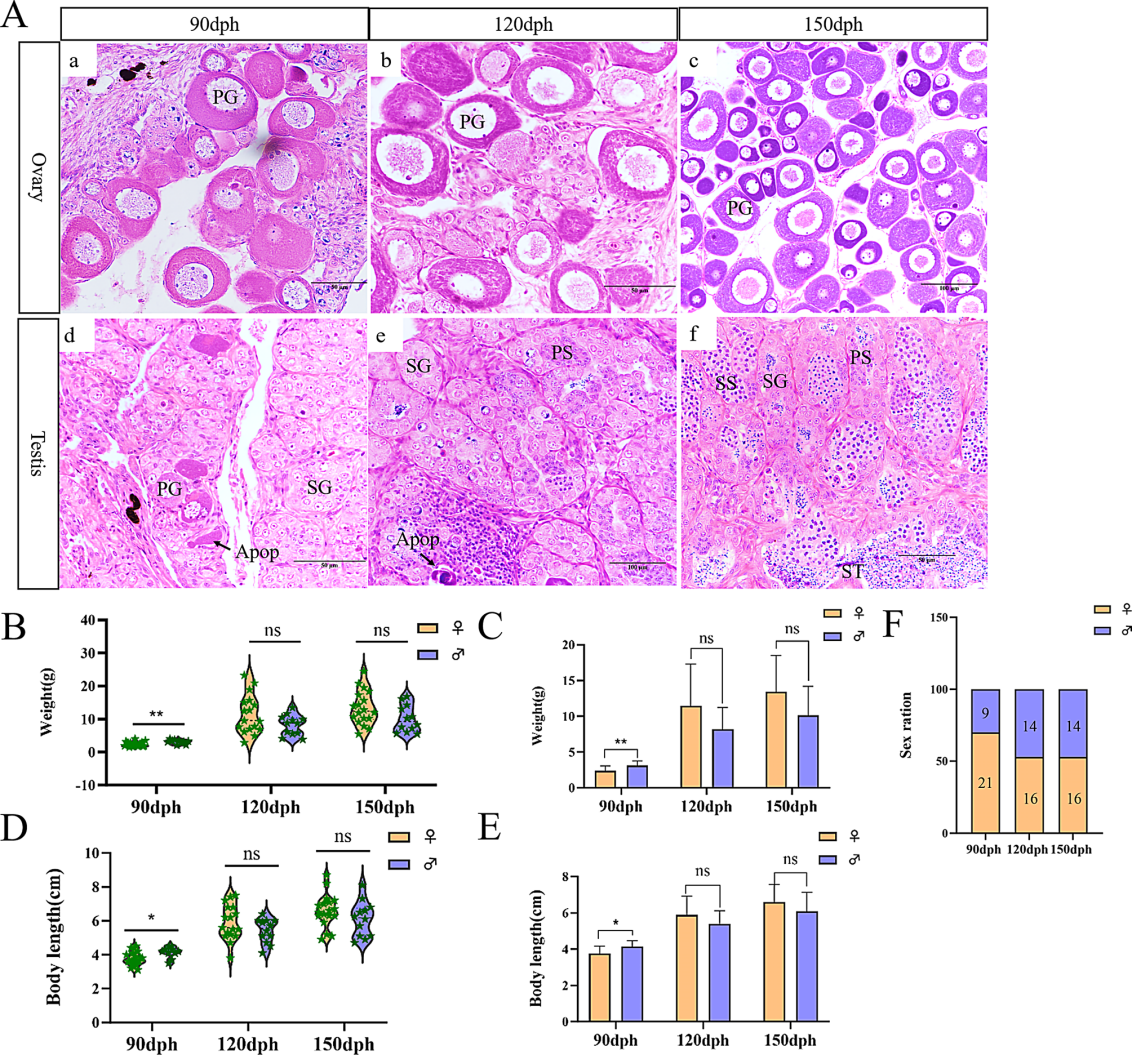
Histological analysis and growth data of silver pomfret at different developmental stages. (**A**) Morphological observation of gonads at various developmental stages in silver pomfret. PG, primary growth oocyte; SG, spermatogonia; PS, primary spermatocyte; SS, secondary spermatocyte; Apop, apoptotic germ cell. (**B**-**E**) Analysis of body metrics across developmental stages, with violin and bar plots illustrating the distribution and differences in body length and weight at different time points, and *p*-values calculated using the non-parametric Mann-Whitney test, *P ≤ 0.05, **P ≤ 0.01. (**F**) Sex ratio at different stages of gonadal differentiation

To characterize sex differentiation and gonadal development stages in silver pomfret, gonad samples were collected at various time points for histological examination. The findings demonstrate that testis formation involves an initial phase of oocyte apoptosis, consistent with previous research [8]. At 90 dph, oocyte apoptosis continued in the testes simultaneously with the presence of oocytes and spermatogonia, indicating juvenile hermaphroditism. At 120 dph, oocyte apoptosis was largely complete, with spermatogonia occupying the entire field of view, though some apoptotic bodies remained visible in the gonads. The process progressed to the testicular development stage, suggesting that 90–120 dph represents the critical period for sex differentiation. By 150 dph, primary spermatocytes (PSs), secondary spermatocytes (SSs), and spermatids (STs) were clearly visible in the testes, while oocytes progressively enlarged throughout development (Fig. 1A) [33, 34]. Sex ratio analysis at different stages confirms 90–120 dph as a crucial period for sex differentiation in silver pomfret. At 90 dph, females significantly outnumbered males, but the sex ratio equilibrated by 120 dph, indicating that some silver pomfret undergo oocyte apoptosis and male differentiation during this period (Fig. 1F). Analysis of body length and weight revealed that females typically exhibit greater body weights than males, with some females showing significantly larger sizes, demonstrating the emergence of sexual size dimorphism during gonadal differentiation (Fig. 1B-E).

### Transcriptome analysis

#### Transcriptome assembly, annotation, and quality assessment

The sequencing analysis generated 848,291,728 raw reads across 18 libraries, yielding 785,081,422 clean reads (92.55%). The data demonstrated high quality with mean Q30 and GC contents of 94.37% and 48.94%, respectively (Table S2), confirming the reliability and suitability of the sequencing data for subsequent analysis. The GC content exhibits species-specific characteristics, so we monitored the AT and GC contents throughout the sequencing process. The distribution results revealed no significant difference between GC and AT content proportions during ovary development. In contrast, the testis exhibited dynamic fluctuations in GC and AT contents over time. At 90 dph, GC and AT levels were nearly identical. However, after sex differentiation (at 120 dph and 150 dph), AT content significantly exceeded GC content (Fig. S1).

**Fig. 2:**
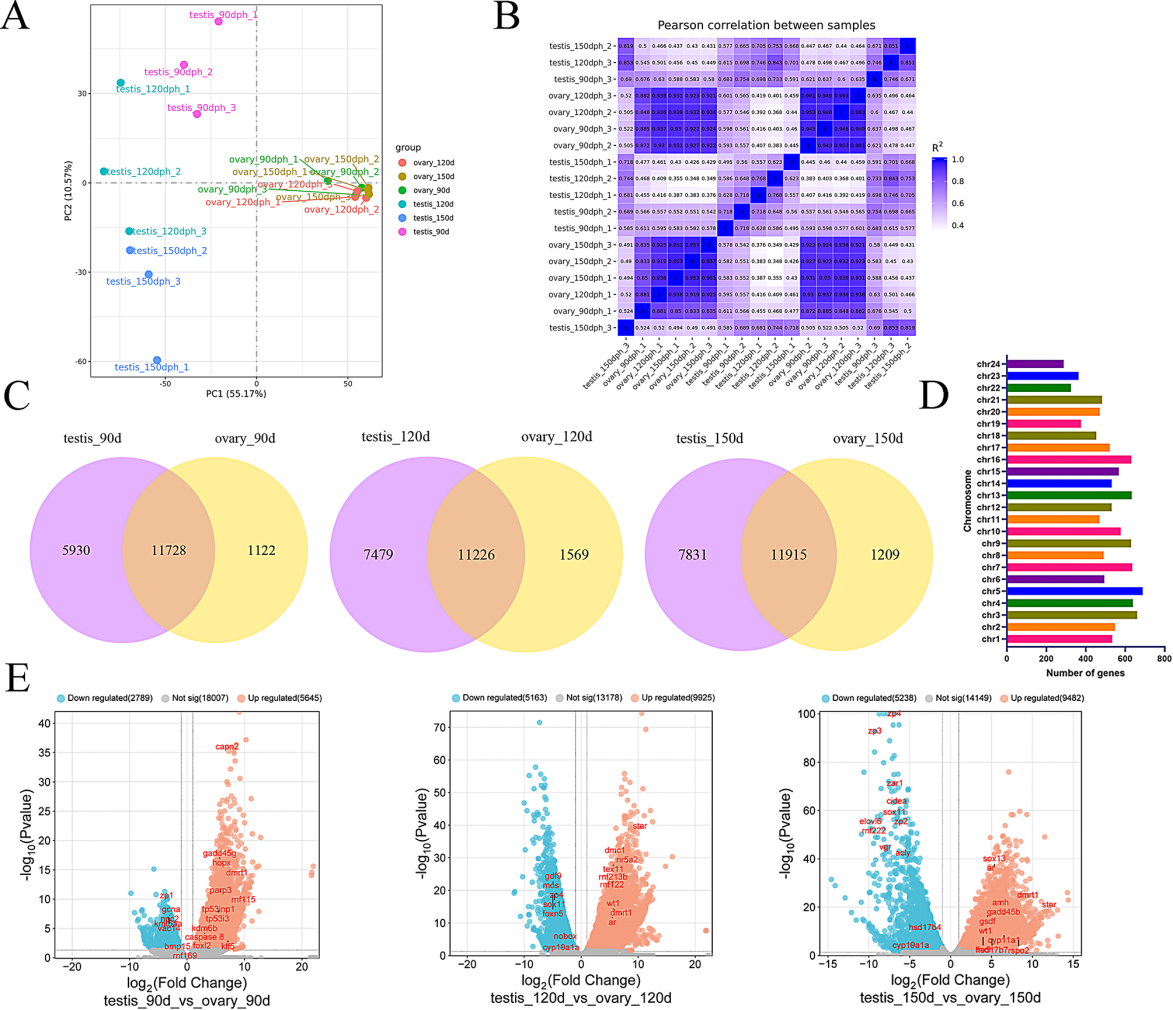
Transcriptomic analysis of gonads in silver pomfret at different developmental stages. (**A**) Principal components analysis (PCA) of gonads from different groups. (**B**) Pearson’s correlation coefficients between different samples. (**C**) Venn diagrams of unigenes annotated at different stages between ovary and testis. (**D**) Distribution of sex differentiation-related genes across different chromosomes. (**E**) Number of DEGs between testes and ovaries at various stages

Gene expression analysis involved mapping all clean reads to the silver pomfret reference genome. The analysis revealed higher total and unique mapping rates in the ovary compared to the testis (Table S3), potentially attributable to the female origin of the reference genome. Analysis of read distribution across genomic regions revealed distinct patterns between testis and ovary tissues. Ovarian tissue consistently maintained exonic region mapping rates exceeding 70%. Conversely, the testis exhibited a gradual decrease in exonic region mapping rates during differentiation, declining from over 70% during early sex differentiation to approximately 60% post-differentiation (Fig. S2). To analyze the transcriptome data variability, Principal components analysis (PCA) was performed, with the first two principal components accounting for 55.17% and 10.57% of the variance between samples, respectively, distinguishing between testes and ovaries (Fig. 2A). The correlation heatmap demonstrated that biological replicates within each group exhibited clustering patterns, indicating the data’s suitability for further analysis (Fig. 2B). Additionally, the co-expression Venn diagram showed minimal variation in co-expressed genes between testis and ovary at different time points. However, during gonadal differentiation, the number of genes expressed exclusively in either testis or ovary increased markedly (Fig. 2C).

#### Identification of DEGs

To investigate genes associated with sex preference in silver pomfret, differentially expressed genes (DEGs) were analyzed in testis and ovary across various developmental stages. The number of DEGs showed significant variation at different time points during sex differentiation (Fig. 2E). At 90 dph, during the initial stage of sex differentiation, 5,645 genes showed upregulation, while 2,789 genes were downregulated. The number of DEGs reached its maximum at 120 dph, with 9,925 upregulated and 5,163 downregulated genes. This peak indicates a critical phase in sex differentiation, aligning with histological observations, and establishes it as a focal point for subsequent analysis. By 150 dph, although sex differentiation was complete, genes associated with gonadal differentiation maintained high expression levels, with 9,482 upregulated and 5,238 downregulated genes. The distribution analysis of DEGs on chromosomes during the critical period of sex differentiation (120 dph) revealed that sex-specific DEGs were distributed throughout the genome (Fig. 2D). These results indicate that sex differentiation in silver pomfret involves regulation by multiple genes.

#### GO and KEGG functional enrichment of DEGs

**Fig. 3:**
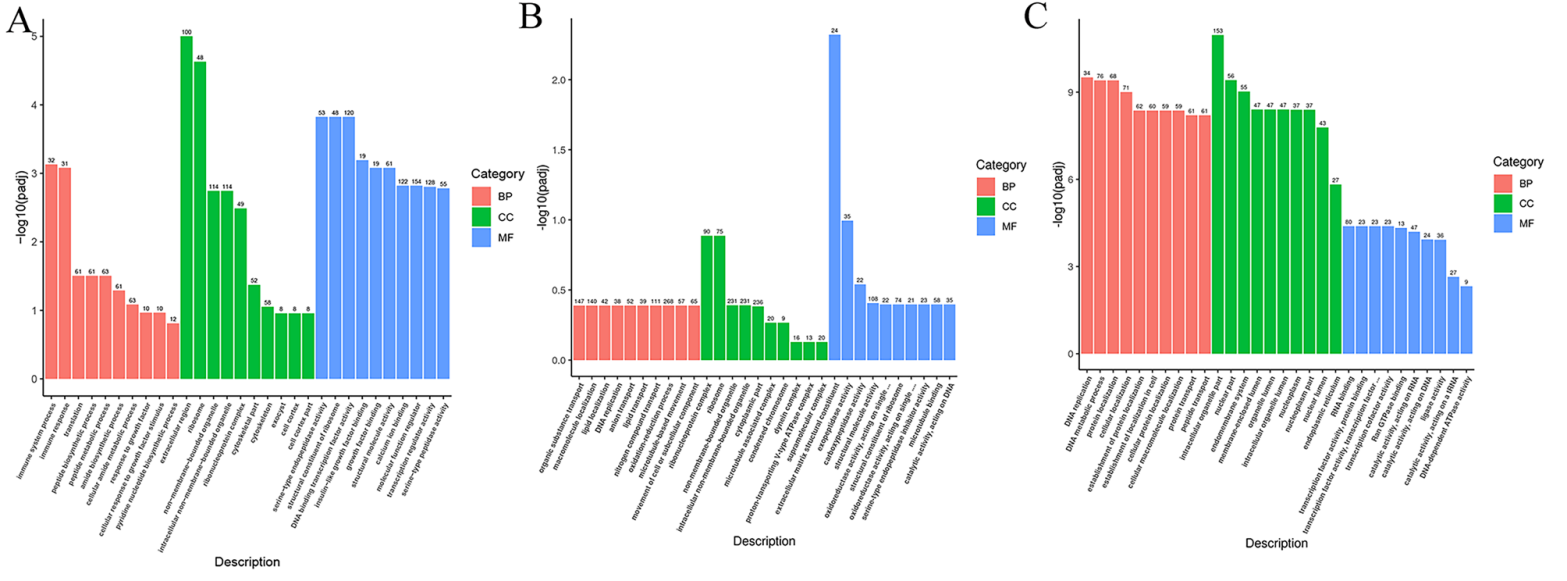
GO analysis of DEGs in 90 dph (**A**) testes, 120 dph (**B**) testes, and 120 dph (**C**) ovaries, highlighting DEGs enriched in the three main GO categories: biological process, cellular component, and molecular function. GO functional enrichment was considered significant with a *padj* < 0.05 as the threshold for significance

In our investigation, DEGs showed significant enrichment across the three primary Gene Ontology (GO) categories: biological process (BP), cellular component (CC), and molecular function (MF) (Table S4). At 90 dph, early-stage oocyte apoptosis occurred in male fish, warranting particular attention. DEGs related to immune system processes (GO:0002376), ribosomes (GO:0005840), insulin-like growth factor binding (GO:0005520), calcium ion binding (GO:0005509), and transcription regulator activity (GO:0140110) showed notable enrichment (Fig. 3A). At 120 dph, during the critical stage of gonadal differentiation, DEGs associated with ribosomes (GO:0005840), the troponin complex (GO:0005861), receptor ligand activity (GO:0048018), and serine-type endopeptidase activity (GO:0004252) were enriched in the testes. Concurrently, during ovary development at 120 dph, DEGs related to protein transport (GO:0015031), endoplasmic reticulum (GO:0005783), RNA binding (GO:0003723), transcription factor activity, protein binding (GO:0000988), ligase activity (GO:0016874), and DNA-dependent ATPase activity (GO:0008094) exhibited significant enrichment (Fig. 3B and C).

**Fig. 4:**
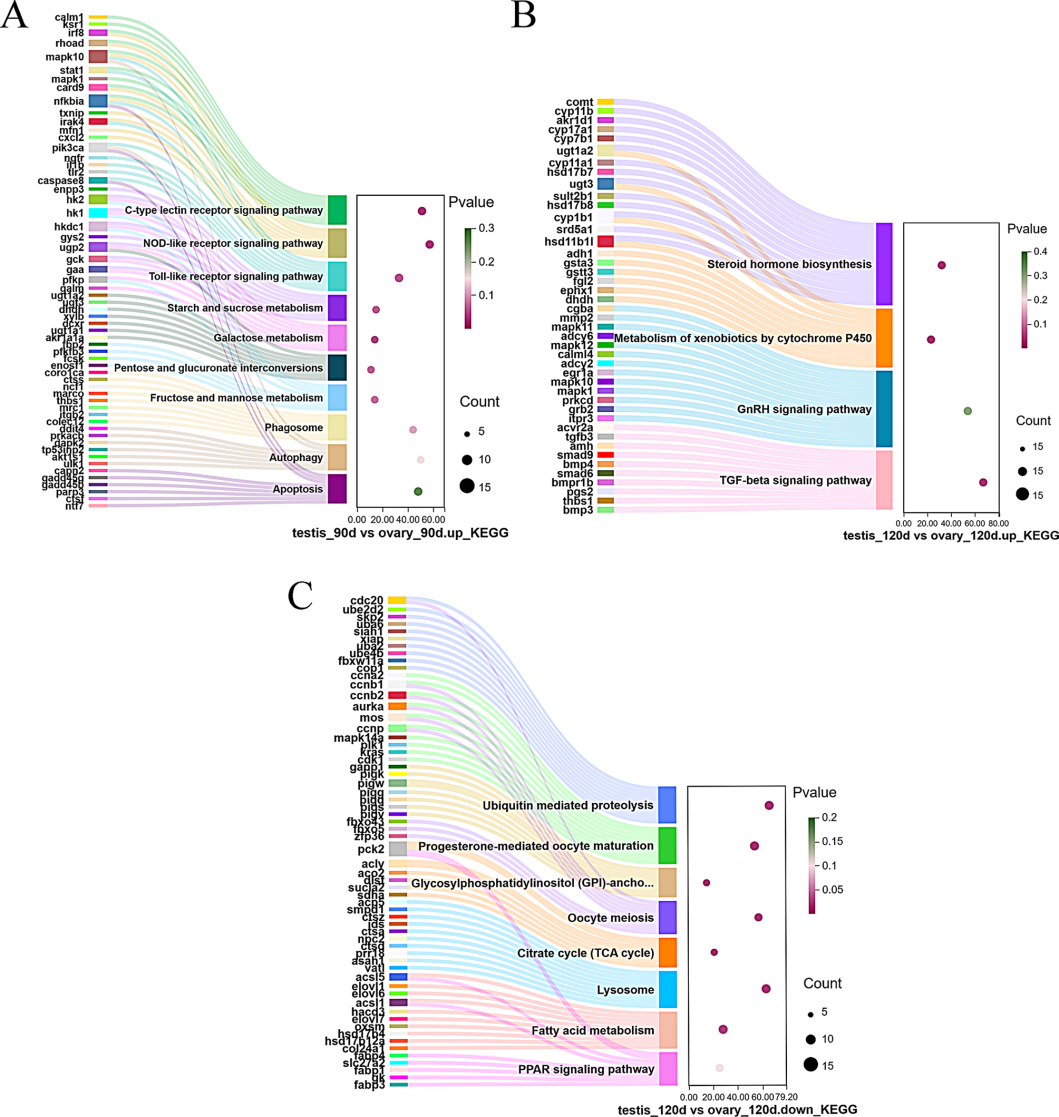
KEGG analysis of DEGs during testis and ovary differentiation at different time points. A *p*-value < 0.05 was used as the threshold for significant enrichment

Regarding KEGG pathway enrichment analysis, Fig. 4A/B/C and Table S5 illustrate the significant pathways involved in silver pomfret gonadal differentiation. The DEGs observed in testes at 90 dph showed significant enrichment in several KEGG pathways, including the C-type lectin receptor signaling pathway (ko04625), NOD-like receptor signaling pathway (ko04621), galactose metabolism (ko00052), Toll-like receptor signaling pathway (ko04620), starch and sucrose metabolism (ko00500), pentose and glucuronate interconversions (ko00040), fructose and mannose metabolism (ko00051), phagosome (ko04145), autophagy-animal (ko04140), and apoptosis (ko04210) (Fig. 4A). During testicular differentiation at 120 dph, DEGs showed notable enrichment in pathways related to steroid hormone biosynthesis (ko00140), metabolism of xenobiotics by cytochrome P450 (ko00980), and the Gonadotropin-releasing hormone (GnRH) signaling pathway (ko04912) (Fig. 4B). Additionally, DEGs associated with ovarian development at 120 dph demonstrated prominent enrichment in KEGG pathways including the ubiquitin-mediated proteolysis (ko04120), progesterone-mediated oocyte maturation (ko04914), glycosylphosphatidylinositol (GPI)-anchor biosynthesis (ko00563), oocyte meiosis (ko04114), the citrate cycle (TCA cycle) (ko00020), the lysosome (ko04142), fatty acid metabolism (ko01212), and the PPAR signaling pathway (ko03320) (Fig. 4C). These pathways serve essential functions in the sex differentiation process of silver pomfret.

### Heatmap analysis of DEGs and validation by RT-qPCR

**Fig. 5:**
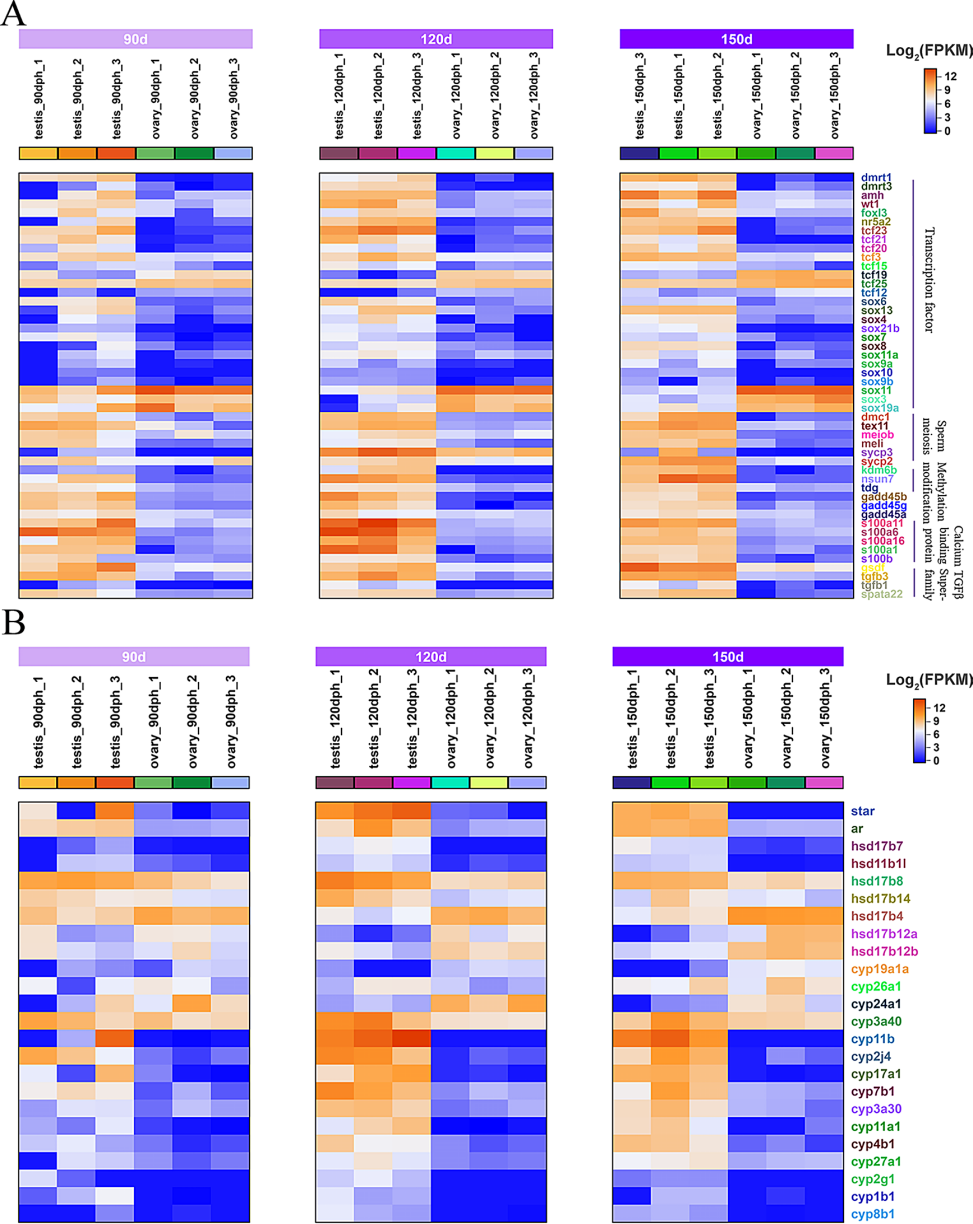
Heatmap analysis of DEGs at different stages in testis and ovary. (**A**) shows heatmap analysis of DEGs including transcription factors, sperm meiosis, methylation modification, calcium-binding proteins, and the TGF-β superfamily. (**B**) shows heatmap analysis of DEGs related to steroid synthesis

**Fig. 6:**
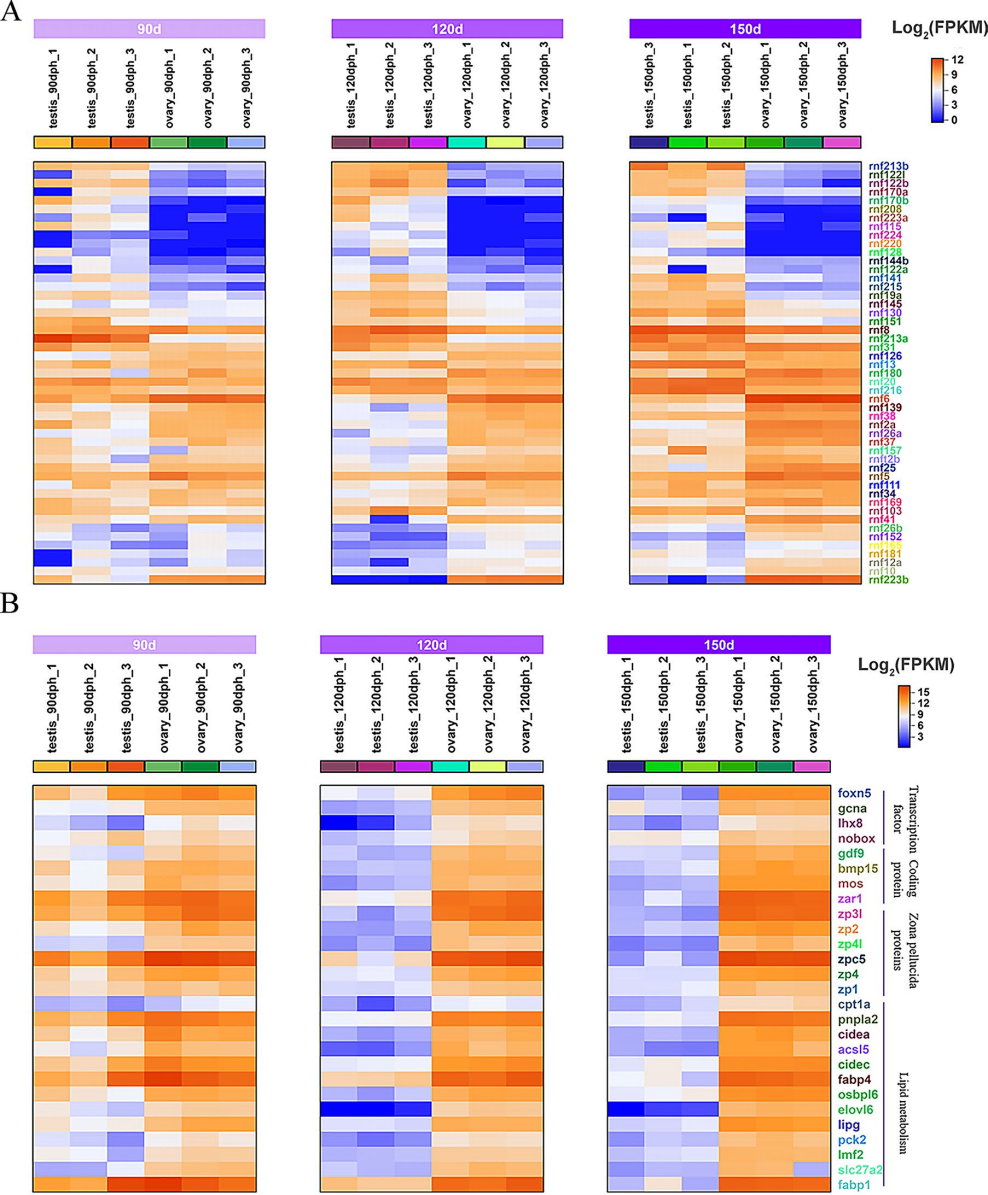
Heatmap analysis of RNFs (**A**) and ovary development-related DEGs (**B**) at different developmental stages in testis and ovary

To examine the expression patterns of sex differentiation genes during the juvenile hermaphroditic phase, we analyzed relevant DEGs and generated expression heatmaps. These DEGs encompass diverse functions, including gene transcription (*dmrt1*; *wt1* [Wilms’ Tumor 1], *foxn5* [forkhead box N5], etc.), spermatocyte meiosis (*dmc1* [disrupted meiotic cDNA 1], *sycp3* [synaptonemal complex protein 3], etc.), steroid synthesis (*ar* [Androgen Receptor], *cyp19a1a* [cytochrome P450 family 19 subfamily A member 1], *hsd17b7* [hydroxysteroid 17-beta dehydrogenase 7], etc.), oocyte development (*zar1* [zygote arrest 1], *zp3* [zona pellucida glycoprotein 3], *zp4* [zona pellucida glycoprotein 4], etc.), mitochondrial energy metabolism (*cox5b*, *cox6a*), and protein ubiquitination (RING Finger Proteins, RNFs) (Figs. 5 and 6). The analysis demonstrated that expression patterns of sex differentiation-related DEGs at 120 and 150 dph exhibited similarity but differed from those at 90 dph. At 90 dph, several genes associated with testicular differentiation had initiated expression, while genes linked to ovarian differentiation were not concurrently downregulated (Figs. 5A and 6B). Concurrently, apoptosis-related genes were significantly enriched (Fig. 9A). To validate the transcriptome data reliability, selected DEGs were quantified in testes and ovaries at various developmental stages using RT‒qPCR. The results confirmed that the expression patterns of all tested genes during gonadal differentiation exhibited sex-specific bias, aligning completely with the RNA-seq findings (Fig. S3 and S4A). These results validate the robustness of the transcriptome data and establish a foundation for identifying key genes in silver pomfret sex differentiation.

### Dynamic expression of genes related to mitochondrial metabolism during sexual development

**Fig. 7:**
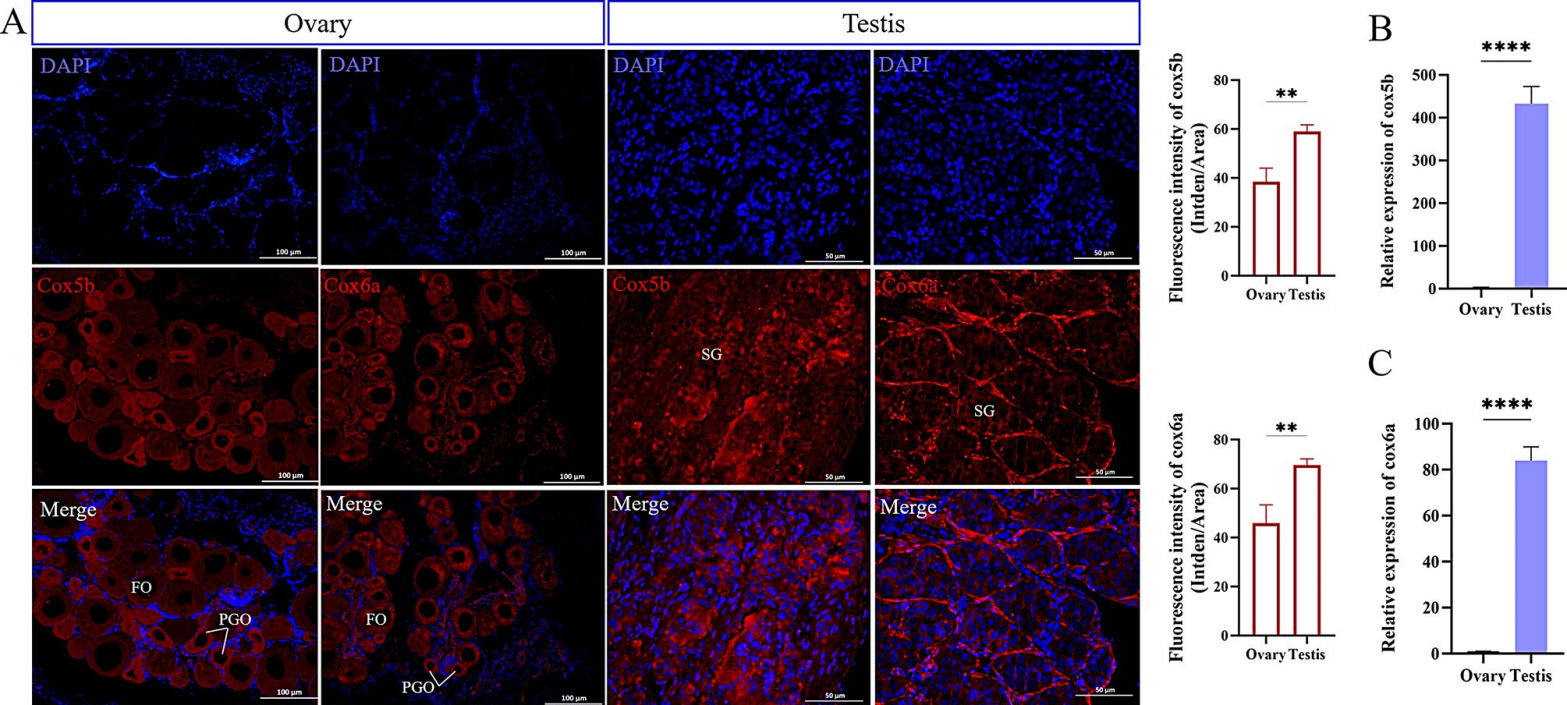
(**A**) Immunohistochemical localization of Cox5b and Cox6a in testis and ovary at 120 dph, with fluorescence intensity quantified using Image J. (**B**/**C**) RT-qPCR analysis of *cox5b* and *cox6a* in testis and ovary at 120 dph. The RT-qPCR results were calculated using the 2^−△△CT^ method with *β-actin* as an internal reference gene and ovary as the control; Values represent means ± standard deviations (*n* = 3); **P ≤ 0.01, ***P ≤ 0.001, and ****P ≤ 0.0001; Statistical analysis was conducted using Tukey’s multiple comparison test

RNA-seq analysis identified significant upregulation of mitochondrial energy metabolism genes in testes compared with ovaries (Fig. 9B). Cox5b and Cox6a, were selected for localization and quantification studies at 120 dph. Western blot analysis confirmed the specific immunoreactivity of both Cox5b and Cox6a antibodies in silver pomfret gonads (Fig. S4B). Immunohistochemistry and RT‒qPCR demonstrated significantly higher expression of both genes in the testes than in the ovaries (Fig. 7A, B and C). Cox5b expression was predominantly observed in germ cells within the testes, while Cox6a was mainly expressed in surrounding somatic cells. In ovaries, Cox5b expression was primarily detected in primary growth oocytes (PGOs), and Cox6a was expressed in both PGOs and follicular oocytes (FOs) (Fig. 7A).

### Differences in sex hormone levels in male and female fish serum during different developmental stages

**Fig. 8:**
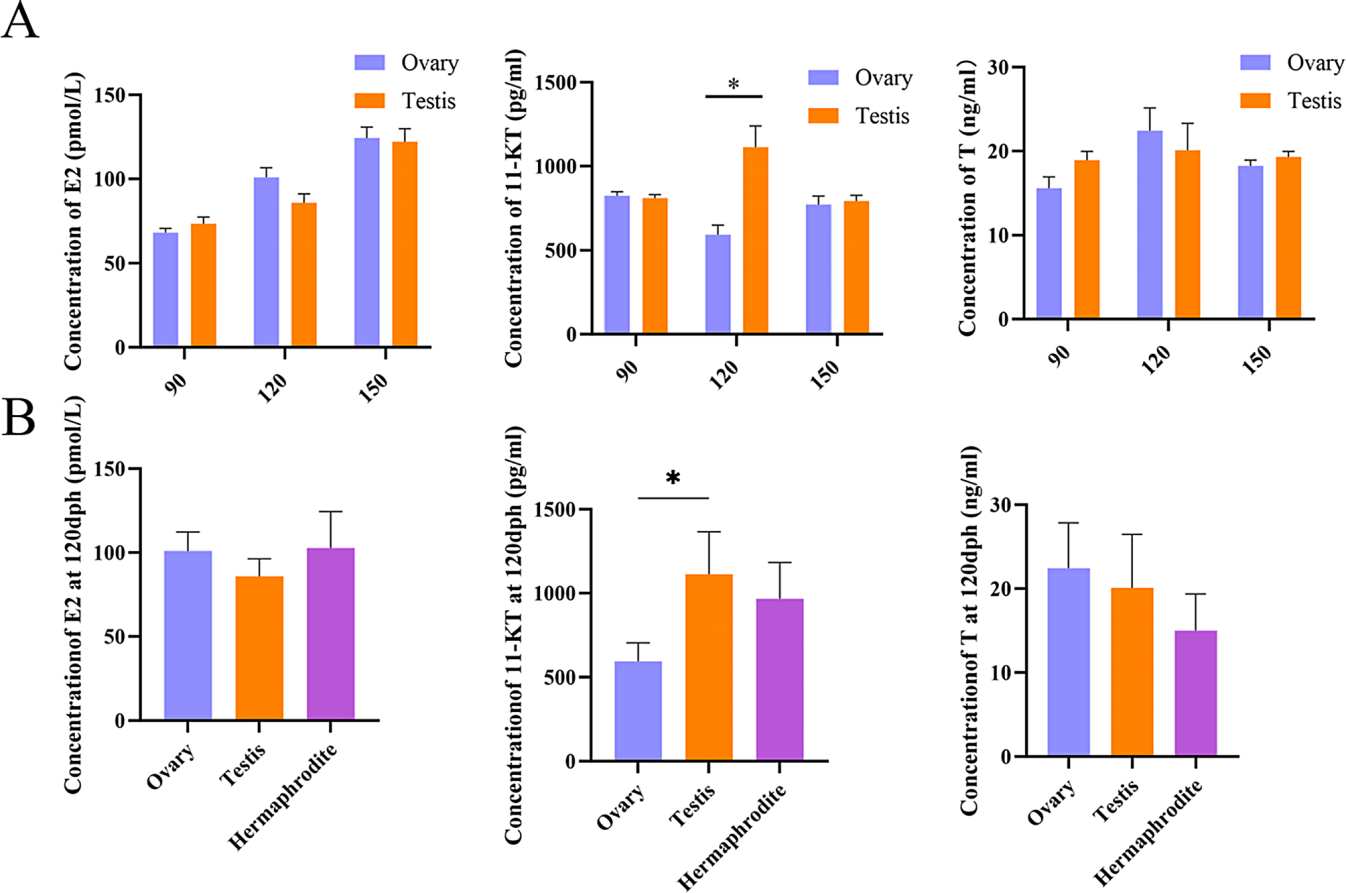
(**A**) Analysis of E2 (17β-estradiol), 11-KT (11-keto-testosterone), and T (testosterone) levels in female and male fish serum at 90, 120 and 150 dph (*n* = 6). (**B**) Measurement of E2, 11-KT and T levels in female, male, and hermaphrodite fish serum at 120 dph (*n* = 6). Statistical differences between two groups with normal distribution and homogeneous variance were evaluated using an independent sample t-test, while comparisons among more than two groups were analyzed using one-way ANOVA, with significance set at *P < 0.05

To examine the potential roles of sex hormones in silver pomfret sex differentiation, the levels of key hormones—E2, 11-KT, and T—were quantified in testes and ovaries at 90, 120, and 150 dph using FISH ELISA kits. Additionally, hormone levels were compared among females, males, and hermaphrodites at 120 dph to investigate their associations with juvenile hermaphroditism. The results revealed that E2, 11-KT, and T levels showed no significant differences between testes and ovaries at 90 dph and 150 dph (Fig. 8A), suggesting potential hormonal regulation overlap during these stages. However, at 120 dph, distinct hormonal profiles emerged, coinciding with critical sex differentiation phases (Fig. 8B). E2 levels were slightly lower in males compared to females and hermaphrodites, indicating a potential role in ovarian development or hermaphroditic trait maintenance. Conversely, 11-KT levels were significantly lower in females compared to males and hermaphrodites, suggesting involvement in testicular differentiation or male-specific development. Notably, testosterone levels were lowest in hermaphroditic fish, suggesting a unique hormonal balance that may facilitate male and female reproductive tissue coexistence during this transitional phase. The data are expressed as the means ± standard deviations (SDs). Statistical differences between two groups with normally distributed data and homogeneous variance were assessed using an independent sample t-test, whereas comparisons among more than two groups were analyzed via one-way ANOVA, with statistical significance set at *P < 0.05.

**Fig. 9:**
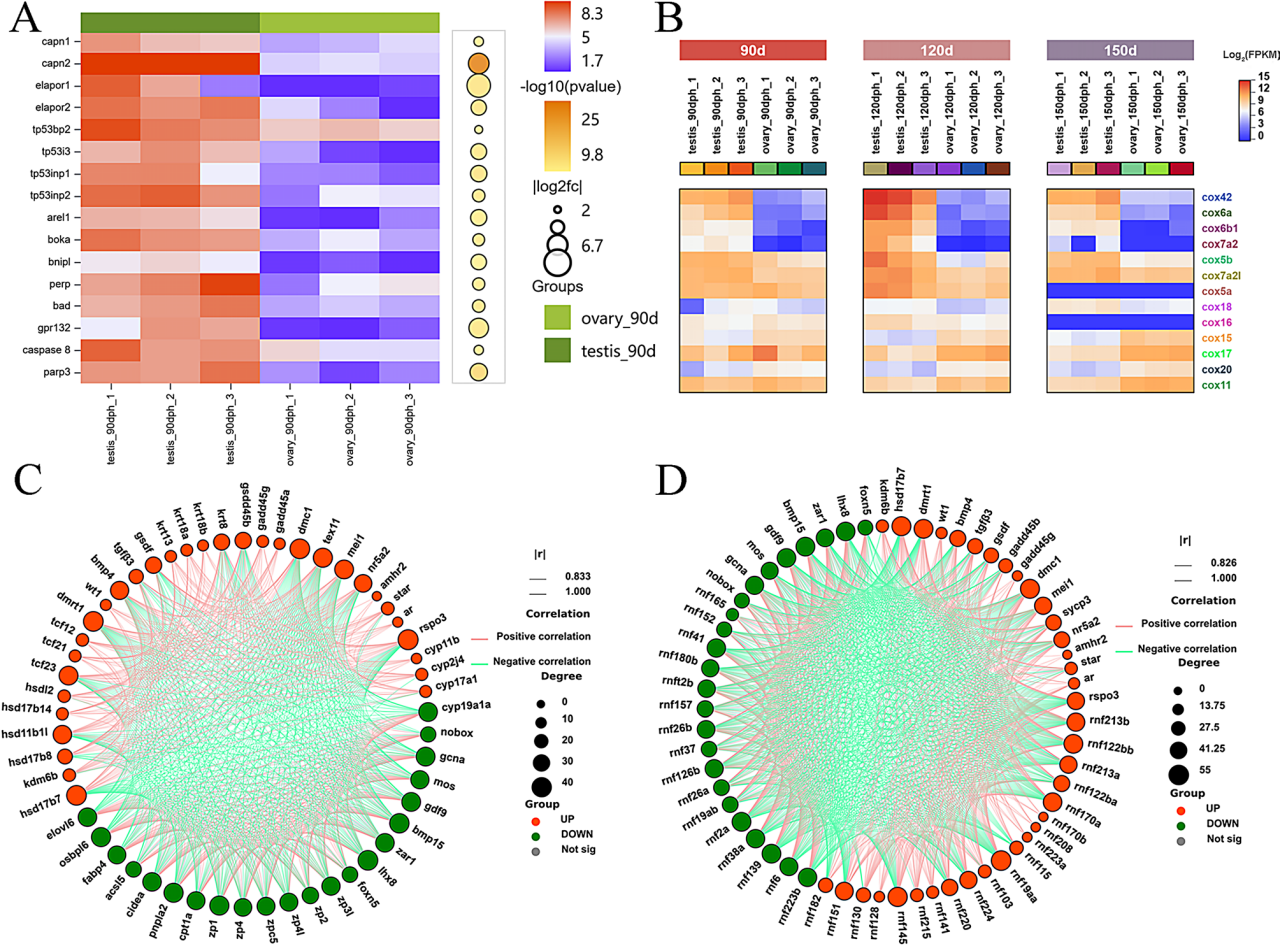
(**A**) Heatmap analysis of apoptosis-related DEGs in testis and ovary at 90 dph; (**B**) Heatmap analysis of COX family members in testis and ovary across different developmental stages; (C/D) Pearson correlation analysis of sex differentiation DEGs

## Discussion

Fish display diverse patterns of sex differentiation, encompassing all known gonadal differentiation patterns found in nature. Among these, juvenile hermaphroditism represents a distinctive type characterized by the initial differentiation of all individuals into ovarian-like gonads during early gonadal development. In genetic females, these gonads develop into functional follicles, while in genetic males, oocytes undergo apoptosis, leading to testis formation. The zebrafish [9], Black Tetra [11], little yellow croaker [10], and silver pomfret examined in this study exhibit this pattern. Silver pomfret demonstrates a longer duration of this stage and more pronounced oocyte apoptosis compared to other species with juvenile hermaphroditism [8]. Furthermore, silver pomfret an economically significant fish species with broad distribution and successful large-scale aquaculture breeding, serves as valuable material for studying fish sex differentiation. Our histological and transcriptomic analyses revealed that the period between 90 and 120 dph was crucial for testis differentiation in silver pomfret. During this stage, oocyte apoptosis occurs in certain gonads while spermatogonia begin to differentiate, resulting in juvenile hermaphroditic development. This differentiation process is regulated through coordinated interactions among multiple signaling pathways.

### Changes in the GC content and read distribution suggest the process of testicular differentiation

The GC content exhibits species-specific characteristics. Between 90 and 150 dph, the GC content in silver pomfret testes fluctuated. At 90 dph, during juvenile hermaphroditic development, the testicular GC content closely resembled that of the ovaries. As gonadal differentiation progressed, the GC content in the testes gradually decreased, reaching approximately 40% at 150 dph. High-GC regions are susceptible to methylation, potentially affecting gene expression [35]. Previous studies have demonstrated that environmental variations can induce changes in methylation levels of sex-related gene promoter regions [36, 37], suggesting that sexual differentiation in silver pomfret is subject to environmental regulation. The onset of testicular differentiation in silver pomfret occurred at 90 dph, characterized by minimal differential expression of sex-biased genes, possibly due to high methylation levels inhibiting gene expression. By 120 dph, gene expression abundance peaked, potentially indicating a demethylation event. Additionally, during testicular differentiation, the proportion of reads mapped to exonic regions decreased, while those mapped to intergenic regions gradually increased. This observation may result from testicular differentiation complexity and the use of a female reference genome, leading to imprecise annotation of some testis differentiation-related genes. Notably, the dynamics of GC content and read distribution across genomic regions have not received substantia attention in RNA-seq studies of sex differentiation in other fish species. Further research is necessary to determine whether these phenomena represent common features of sex differentiation across fish species or are specific to silver pomfret.

### A shift in metabolism may be a key factor driving oocyte apoptosis

The apoptosis of early oocytes represents a critical step in testis development. However, current conclusions are based primarily on histological sections and TUNEL staining [8], lacking supporting molecular evidence. In the transcriptome of 90 dph male fish, significant enrichment was observed in genes related to the apoptosis pathway and apoptosis-related genes. These findings confirm that oocyte reduction during the ovary-to-testis transition in juveniles occurs through apoptosis, supporting previous conclusions. Histological results demonstrated extensive apoptosis of early oocytes in male fish at 80–90 dph [8]. To maintain normal physiological functions, these apoptotic oocytes require prompt clearance, resulting in significant enrichment of phagosome and autophagy processes. Furthermore, transcriptomic analysis of 90-dph male fish revealed significant enrichment of immune pathways, including the C-type lectin receptor signaling pathway, NOD-like receptor signaling pathway, and Toll-like receptor signaling pathway via KEGG analysis. The immune system process showed notable enrichment in GO analysis. These findings suggest that extensive oocyte apoptosis may trigger an inflammatory response, subsequently inducing an immune response. Additionally, ovary development typically involves lipid accumulation, whereas testis differentiation requires minimal lipids [8]. At 90 dph, lipid metabolism pathways showed no significant enrichment in the testes, potentially contributing to oocyte apoptosis rather than continued development. Conversely, carbohydrate metabolism pathways, including starch and sucrose metabolism, galactose metabolism, pentose and glucuronate interconversions, and fructose and mannose metabolism, demonstrated significant enrichment during this period. Moreover, members of the cytochrome c oxidase family, crucial enzyme complexes in the mitochondrial respiratory chain essential for oxidative phosphorylation and ATP production, showed high expression in the testes. Sperm cells contain numerous mitochondria that depend on cytochrome c oxidase for efficient electron transfer [38]. This observation was further supported by immunohistochemistry and qPCR results for *cox6a* and *cox5b* in the gonads. These findings indicate that during critical testis differentiation, energy supply primarily derives from carbohydrate metabolism. Additionally, other studies have demonstrated that testicular development heavily relies on glucose metabolism within Sertoli cells [39].

### The simultaneous high expression of genes related to testis and ovary differentiation is the fundamental reason for juvenile hermaphroditism

At 90 dph, nonapoptotic oocytes coexisted with differentiated spermatogonia, a phenomenon termed juvenile hermaphroditism. Transcriptomic analysis demonstrated that, despite the initiation of oocyte apoptosis at this stage, the expression of key ovarian development genes, including *foxn5* [40], newborn ovary homeobox gene (*nobox*) [9], growth differentiation factor 9 (*gdf9*) [41], and bone morphogenetic protein 15 (*bmp15*) [41], remained significantly expressed. Concurrently, genes associated with testis differentiation, such as *dmrt1* [16], *wt1* [42], *gsdf* [18], anti-Müllerian hormone (*amh*) [16, 43], and *dmc1* [44], had already begun to exhibit high expression. This pattern indicates the simultaneous high-level expression of both testis and ovary differentiation genes during the juvenile hermaphroditic stage. In contrast, other fish species, exhibit antagonistic interactions between these genes that determine gonadal differentiation. In tilapia, germ cell fate is regulated by the antagonistic interaction between the testis-determining gene *dmrt1* and the ovary-determining gene *foxl3* [45]. In zebrafish, *dmrt1 facilitates* testis differentiation through a dual mechanism that involves transcriptional activation of *amh* and suppression of *foxl2* expression [5]. The knockout of *dmrt1* results in upregulation of ovary-differentiating genes including *cyp19a1a*, leading to ovarian differentiation [46]. In Nile tilapia, *gsdf* functions downstream of *dmrt1* to initiate testis differentiation by inhibiting estrogen production [47]. These observations suggest that the asynchronous regulation between ovary-related gene downregulation and testis-related gene upregulation likely underlies juvenile hermaphroditism.

### The coordinated expression of 11-KT and sex differentiation-related genes promotes testicular development in silver pomfret

Both histological and transcriptome analyses revealed that 90–120 dph represents a critical period for testicular differentiation in silver pomfret. During this stage, certain individuals undergo oocyte apoptosis and spermatogonial development. The number of DEGs in the testes and ovaries increased from 26,441 at 90 dph to 28,266 at 120 dph, reaching its peak at 120 dph, indicating that 120 dph marks a crucial point in sex differentiation. Furthermore, the identification of 7,479 testis-specific genes and 1,569 ovary-specific genes emphasizes the predominance of genes linked to testis development. Previous histological studies have shown that ovarian differentiation begins at 60 dph, with oocytes entering a stable growth phase by 120 dph [8], explaining the comparatively lower number of genes associated with ovarian differentiation. Multiple studies have established that sex hormones serve a crucial role in directing sex differentiation by regulating the expression of specific genes [48]. In our study on silver pomfret, we observed that administering 17α-methyltestosterone (MT) before 90 dph induced oocyte apoptosis in the gonads, accompanied by testicular differentiation [8]. This pattern mirrors the transition observed during the juvenile hermaphroditic stage in silver pomfret, suggesting that sex hormones likely influence sex differentiation in this species. Sex hormones are synthesized primarily from cholesterol [49]. During testicular differentiation of silver pomfret, pathways related to steroid hormone biosynthesis and the metabolism of xenobiotics by cytochrome P450 showed significant enrichment. Moreover, members of the hydroxysteroid (17β) dehydrogenase family (HSD17Bs) and the cytochrome P450 superfamily (CYPs) exhibited differential expression during gonadal differentiation. These pathways and genes are essential for sex hormones synthesis. For instance, *cyp11a1* converts cholesterol to progesterone [50], *cyp19a1a* catalyzes the conversion of testosterone (T) to estradiol (E2) [51], and *hsd17b7* facilitates the conversion of the weaker estrogen estrone (E1) to the biologically more potent estradiol (E2) [52]. Additionally, the GnRH signaling pathway showed significant enrichment during testis differentiation. GnRH, synthesized and released from the hypothalamus, stimulates the anterior pituitary to secrete gonadotropins, including luteinizing hormone (LH) and follicle-stimulating hormone (FSH) [53]. LH promotes androgen synthesis by stimulating Leydig cell activity [54]. This evidence suggests that sex hormones may influence sex differentiation in silver pomfret. Similarly, our findings indicate that there are no significant differences in sex hormone levels between female and male silver pomfret at 90 dph and 150 dph. However, at 120 dph, a marked difference in hormone levels between the sexes was observed. This key finding aligns with histological observations and transcriptomic analyses, confirming that 120 dph represents a critical threshold for sex differentiation in silver pomfret. E2 functions as the primary estrogen in fish, while 11-KT serves as the principal androgen [48]. The distinct expression of E2 in females and the elevated levels of 11-KT in males at 120 dph highlight the central regulatory roles of these sex hormones in sex differentiation. This pattern is particularly evident for the androgen 11-KT, corresponding with observations in other fish species, such as the blackeye goby [55] and little yellow croaker [10].The elevated serum levels of both E2 and 11-KT in hermaphroditic fish may explain why some individuals had not completed sex transition by 120 dph. These findings suggest that manipulating sex hormone levels could enable targeted breeding of monosex populations in silver pomfret.

Sex hormones regulate the expression of sex differentiation genes, many of which have been identified during sex differentiation in silver pomfret. Besides the sex-determining genes *dmrt1* [14], *amh* [17], and *gsdf* [18], several SRY-related HMG-box (SOX) family genes also exhibit significant differential expression during the sex differentiation of silver pomfret, particularly at 120 dph. Notably, unlike other gene families, RNFs are seldom associated with sex differentiation processes in fish. RNFs, characterized by a ring finger domain, are crucial for regulating protein ubiquitination and degradation [56, 57], with functions including cell cycle regulation [58], signal transduction [57], and apoptosis modulation [59]. Significantly, RNFs show enrichment during the sex differentiation of little yellow croaker [60], a species exhibiting juvenile hermaphroditism similar to silver pomfret [10]. This observation suggests a potential role for RNFs in regulating gonadal juvenile hermaphroditism, a hypothesis warranting further investigation. Furthermore, Pearson correlation analysis of sex differentiation genes in silver pomfret (Fig. 9C and D) revealed that these genes operate coordinately to regulate gonadal differentiation, emphasizing the complexity of the underlying molecular mechanisms.

### Ovary development involves lipid accumulation and the natural selection process of follicles

Ovarian development requires substantial energy reserves. Studies show that lipid deposition in the ovaries begins at the onset of sex differentiation [8]. As a result, pathways associated with lipid metabolism, including fatty acid metabolism and the PPAR signaling pathway, along with energy metabolism pathways, such as the citrate cycle (TCA cycle), exhibited significant enrichment during ovarian differentiation. Furthermore, key lipid metabolism genes (*cpt1a*, *pnpla2*, *cidea*, *acsl5*, *fabp4*, etc.) demonstrated high expression in the ovaries. Previous research established that follicle development represents a natural selection process wherein nondominant or immature follicles undergo atresia [33]. Ubiquitin-mediated proteolysis and the lysosome likely facilitate protein degradation and organelle clearance, thereby eliminating abnormal follicular development [61, 62]. Additionally, the proper function of pathways including progesterone-mediated oocyte maturation and oocyte meiosis is essential for normal follicular development. Ovarian development involves coordinated interactions between somatic cells (such as follicular granulosa cells and theca cells) and germ cells (oocytes). The GPI-anchor biosynthesis pathway facilitates cell-cell communication [63], which is crucial for proper follicular development.

## Conclusion

In summary, these findings further validate the new model of sex differentiation in silver pomfret. This model demonstrates that apoptotic signals regulate early oocyte apoptosis, while the brief juvenile hermaphroditism phase results from concurrent high expression of testis-preferential and ovary-preferential genes. The research establishes that sex hormones, particularly androgen 11-KT, determine sex differentiation direction by modulating related gene expression. This process encompasses differential expression in key pathways, including steroid hormone biosynthesis, metabolism of xenobiotics by cytochrome P450, the GnRH signaling pathway, and sex differentiation-associated genes. Furthermore, ovarian development incorporates energy accumulation and natural selection among follicles.

## Electronic supplementary material

Below is the link to the electronic supplementary material.

Supplementary Material 1: Figure S1 Distribution of GC and AT content among different samples during sequencing.

Supplementary Material 2: Figure S2 Distribution of reads from different samples in the genomic exonic regions, intronic regions, and intergenic regions.

Supplementary Material 3: Figure S3 Validation of DEGs in testes and ovaries at 90, 120, and 150 dph using RT-qPCR. The RT-qPCR results were calculated using the 2^−△△CT^ method with *β-actin* as an internal reference gene and ovary as the control; Values represent means ± standard deviations (*n* = 3); **P ≤ 0.01, ***P ≤ 0.001, and ****P ≤ 0.0001; Statistical analysis was performed using Tukey’s multiple comparison test.

Supplementary Material 4: Figure S4 (A) mRNA expression levels of apoptosis-related genes in gonadal tissues at 90 dph were detected by RT-qPCR. The data analysis method was the same as in Fig. S3. (B) Specificity validation of Cox5b and Cox6a antibodies in gonadal tissues (Western blot).

Supplementary Material 5

Supplementary Material 6

Supplementary Material 7

Supplementary Material 8

Supplementary Material 9

## Data Availability

Most data generated or analyzed during this study are included in this published article and its supplementary information files. The transcriptome data can be accessed at the National Center for Biotechnology Information (BioProject accession number: SUB14662292).
