# Supplementary figures and images for: Transcriptome analysis reveals the sex-switching mechanism of juvenile hermaphroditism in silver pomfret (Pampus argenteus)
Source: Biol Sex Differ. 2025 Jul 14;16:51. doi: 10.1186/s13293-025-00736-1 (PMC12261592; doi:10.1186/s13293-025-00736-1)

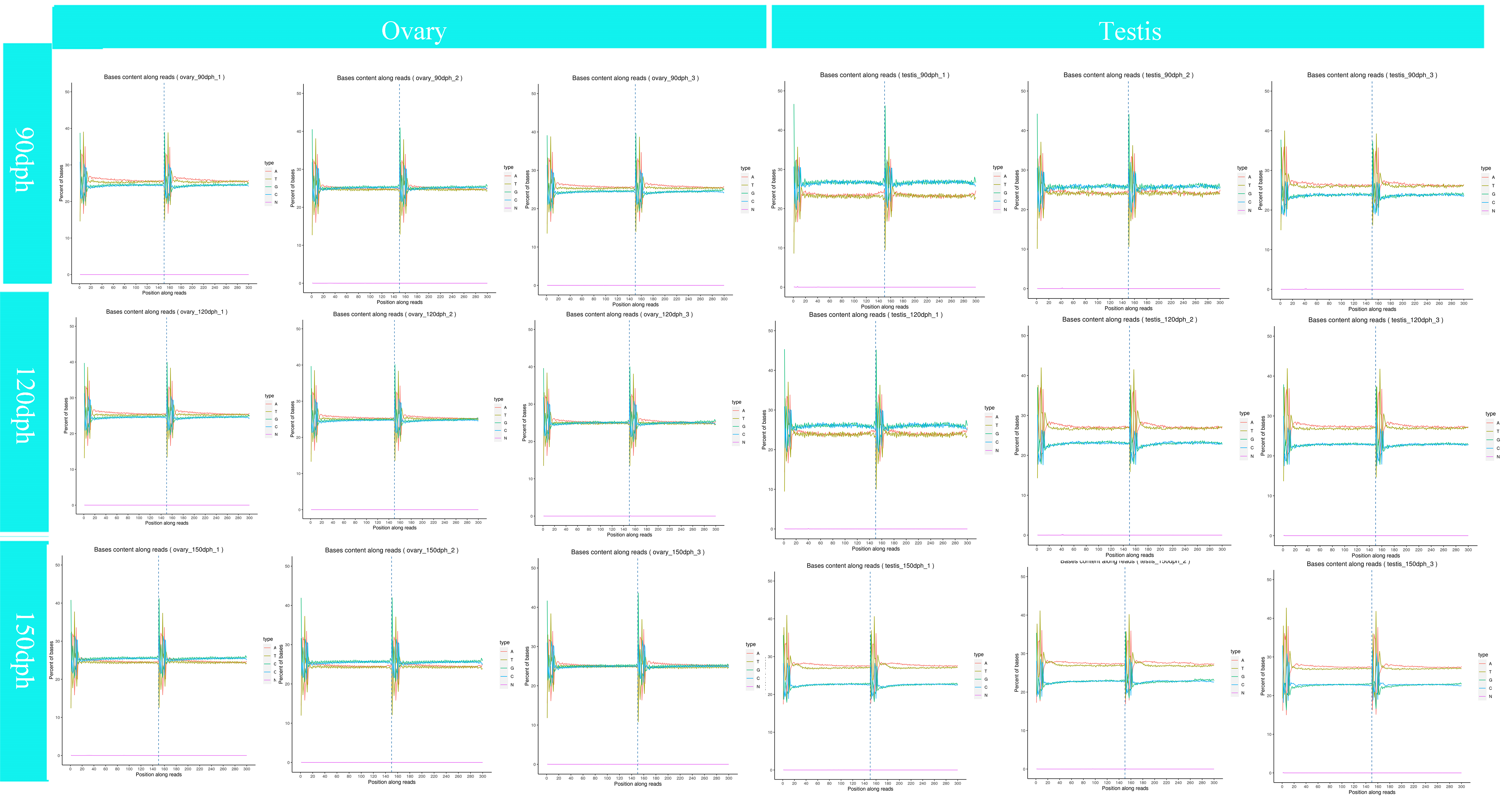

Supplement: Supplementary file 1 — Supplementary Material 1: Figure S1 Distribution of GC and AT content among different samples during sequencing. [file 13293_2025_736_MOESM1_ESM.png]

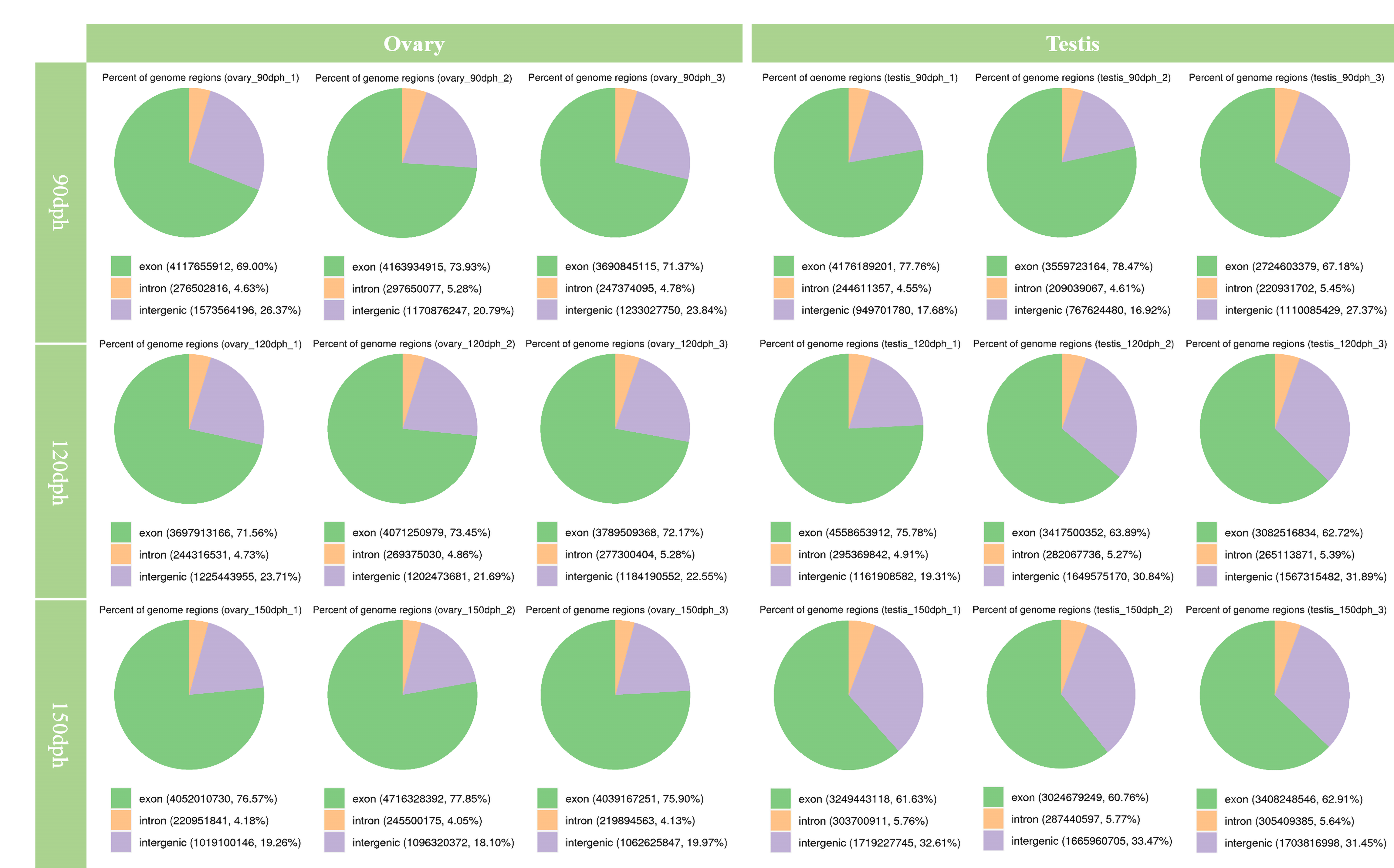

Supplement: Supplementary file 2 — Supplementary Material 2: Figure S2 Distribution of reads from different samples in the genomic exonic regions, intronic regions, and intergenic regions. [file 13293_2025_736_MOESM2_ESM.png]

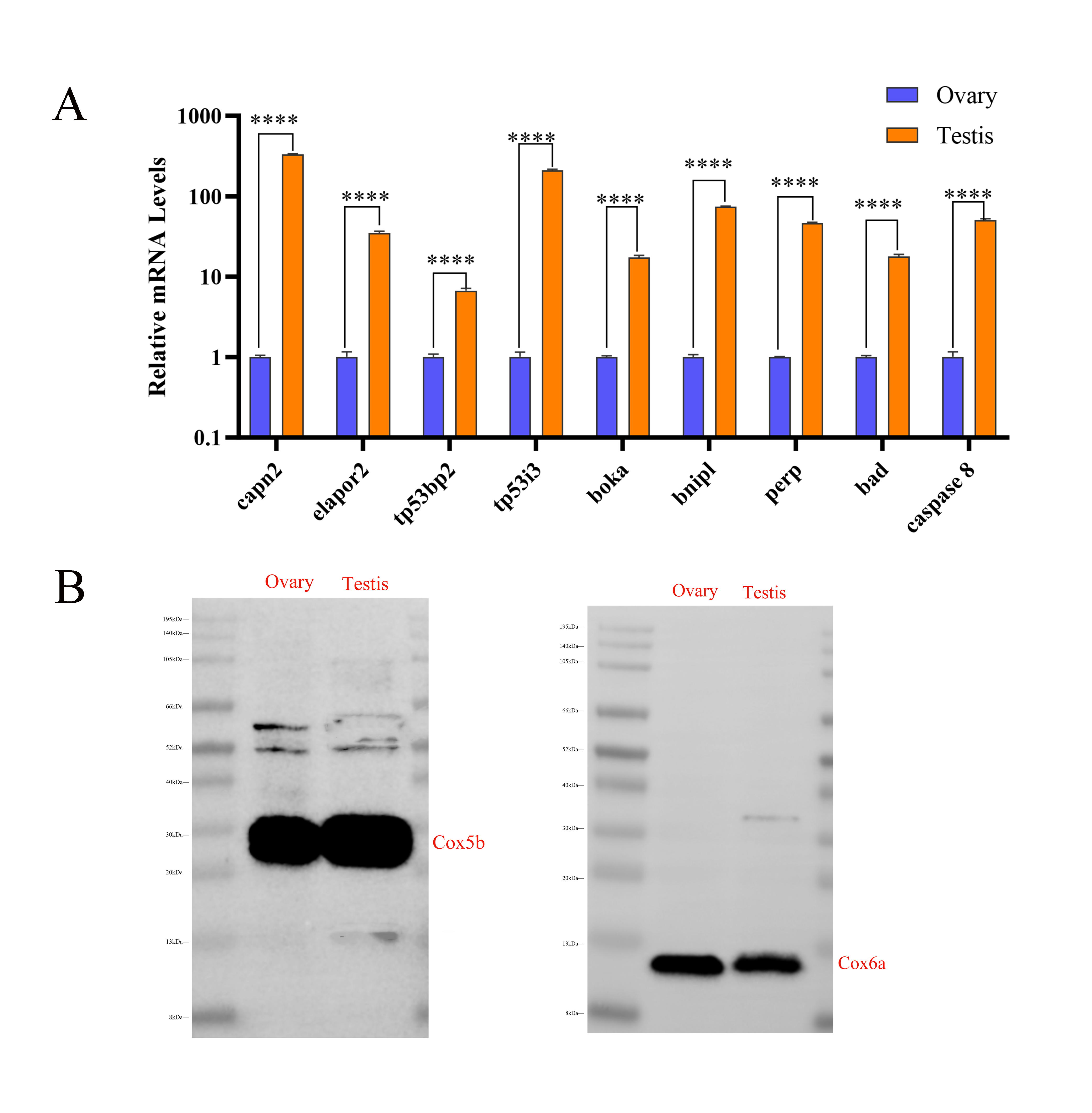

Supplement: Supplementary file 4 — Supplementary Material 4: Figure S4 (A) mRNA expression levels of apoptosis-related genes in gonadal tissues at 90 dph were detected by RT-qPCR. The data analysis method was the same as in Fig. S3. (B) Specificity validation of Cox5b and Cox6a antibodies in gonadal tissues (Western blot). [file 13293_2025_736_MOESM4_ESM.png]
