# Supplementary material for: Transcriptome analysis reveals the sex-switching mechanism of juvenile hermaphroditism in silver pomfret (Pampus argenteus)
Source: Biol Sex Differ. 2025 Jul 14;16:51. doi: 10.1186/s13293-025-00736-1 (PMC12261592; doi:10.1186/s13293-025-00736-1)
Supplement: Supplementary file 5 — Supplementary Material 5 [file 13293_2025_736_MOESM5_ESM.docx]

**Table S1** Primers for real-time quantitative PCR (RT-qPCR)

| Gene name | Forward primer (5’-3’) | Reverse primer (5’-3’) | Product | Tm(℃) |
| --- | --- | --- | --- | --- |
| *β-actin* | TGGCATCACACCTTCTACAAC | ACGACCAGAGGCATACAGG | 121 | 58 |
| *dmrt1* | AGTGTCCCAAATGTAAACTGATAGC | GCGACTCCCTGTAACACCAAAA | 236 | 58 |
| *amh* | CGGAGGCTGGAGCAGATTG | AGAAGAAACGCACGATACTGGCT | 164 | 58 |
| *wt1* | CATAGACGACCCTCACTGTCAACTT | TGACCGTAACTGGGGTTGCTGT | 225 | 58 |
| *nr5a2* | CAGCAGAAGAAGGCTTTGATACG | GAAGGAGACTTTTGGAGATGGTAG | 139 | 58 |
| *tcf23* | CACCTGATACCAAACTGTCCAAG | GATTGGTCGGGACTCCTGATAG | 190 | 58 |
| *tcf20* | AGTAGTTGTGGTAGCAGTGGCAGT | TTGTCAAGTGGAGAAGATGAATAA | 214 | 58 |
| *tcf12* | CAGTCAGTTCAGTGCGTCAGGTA | CATTATGTTCGAGTTCACAAAAGGT | 196 | 58 |
| *sox6* | TAAGCAAGATTTTAGGTTCTCGGTG | TGAGGTGAGGGTGTAGTAGTCGC | 219 | 58 |
| *sox13* | ACCAGGAGAAGCAGCCTTACTAT | GTTCTGTTTGACTGTGCGTGTAG | 204 | 58 |
| *sox9a* | GCGGAGAAAGTCTGTGAAGAACGG | TTCACGCTTTAGGTCCATCTTGC | 229 | 58 |
| *sox9b* | AGATGACAGAAGAACAGGAGAAG | CATCTCTGATACACACGGGAAACT | 199 | 58 |
| *sox3* | ATGGCACAAGAAAATCCCAAAAT | GAGTTGTTGACGGCATTGGCT | 251 | 58 |
| *sox11* | CCCAAAAAGAAGCCCAAGACTG | GTTTACTGGGTTTCAACTTGGAGA | 145 | 58 |
| *dmc1* | CTGGGAAAATGCTGAGTGTTGGT | CATCTGGTGTTCGCTGGTGTAGG | 203 | 58 |
| *meiob* | ACTCAGTCTCTTCGTTGCCCCT | CCAGACTCAGACACCTCTTTAGCA | 232 | 58 |
| *meli* | GAAGTCTTTTGCCTTGTCTGGTATC | CAAAACAAAGCGAAATGACAACTC | 184 | 58 |
| *sycp3* | GGGAGACTGAAGCCCAGCGAGC | GCTGTTGCGTGTCCATGAGGATCTT | 258 | 58 |
| *kdm6b* | ATCAAGATCACCGACCAGGATAC | GATACTGTTCAAGCACCACGACT | 166 | 58 |
| *s100a11* | TGACTGACTTGGAGAAATGTATGG | CAGTCCACAGTTTTGGGGTTATT | 120 | 58 |
| *gsdf* | CATCCTGCCAGCCTTACCATCG | CTGGGAGGTGGGCTGACAACAT | 138 | 58 |
| *tgfβ3* | GCTCTTCTGTTTTTCCTCCTCTCA | TGAGCTCCTTGGTGCTGTTATAAA | 217 | 58 |
| *spata22* | GCACCAACCGTATGACAGTAGAC | CAGTATGAGGTGTTTGCTTGCTTT | 227 | 58 |
| *hsd17b7* | CGTCTGGACTACCTCTACCTGAAT | GATTAGTAGAAAATGACCGAAGAG | 207 | 58 |
| *hsd11b1la* | CCTTCAGTATGTGGGATGGAGAT | TTACCTAAAAGTGATGAAACAACCA | 148 | 58 |
| *hsd11b1* | CACGAGTTGGCTATGAAGAAGAG | GTTGTAGTTGTAGGAGTTTTGGATGA | 267 | 58 |
| *cyp11b* | GAGGAGGATTGCTGAAAATGAGAT | GGTGTATGTGGTTTTGATGTCTTCT | 103 | 58 |
| *cyp2j4* | TCAATGAAACTAATCTGGCTATGTG | GGATTTCAGGGTTTTTGATAAGG | 114 | 58 |
| *cyp17a1* | AAGGTCTTTCCTAACAAGTCTCTCA | CTGTGATCTCCCCATCCTCTAAC | 202 | 58 |
| AR | CAGGCTGAAAGAACACGACAAT | CCTGGCTGTTTCTGAGATTGTG | 196 | 58 |
| *hsd17b4* | CTCAATCCCTGGACTCAACATAG | CTCCAACGACAAACACTGAAAAC | 221 | 58 |
| *hsd17b12a* | ACAGGCGTCAGGGTATCATCAT | TCACTCTTCAGACTCGCTCCGT | 213 | 58 |
| *cyp19a1a* | ACTGTACCAGGTCCTTCTTTCTTT | AACGTGAAACGTAATGTCCATGTT | 211 | 58 |
| *rnf213b* | TGACCTCTCTCCTGCCTGGATAAT | AACAAATGACTCACACTCTTGCTGG | 229 | 58 |
| *rnf170* | GTGTCAGCAACCAGGTCTTATTT | GAACCACAGAAAAGATGTCCACAAT | 271 | 58 |
| *rnf122b* | AGCCTCATCTTCTGCTGCTACTTG | CTGCGAAACTCCTCTAAACACACC | 149 | 58 |
| *rnf19a* | CGCTTATGTTGACCTATGTCTACG | TGTCATTGAGTGTTGGGTTGTTCT | 186 | 58 |
| *rnf6* | GGAGCATAATGACACCTCGGATAC | GGCTCATAGAAAGAGTGGTAGTGC | 209 | 58 |
| *rnf139* | GCCATTTTCAACTCTTACTACGACC | CACCACACTGGAGATGAGGACAC | 99 | 58 |
| *foxn5* | CAGAAGAAATCCGAACGAGTCC | TTACTGTCCCTGACTTTTATGGTTC | 190 | 58 |
| *gcna* | AATACCAGAGCGTGGGAGTTTAG | AAGTGCTCTTGACCACAATGTTATC | 221 | 58 |
| *lhx8* | AAGAAGAGTTGGACAAAGATGGC | GTTCTGAAACCAAACCTGAATGAC | 197 | 58 |
| *bmp15* | TCCTCTTCCTCCTCCTCCTCTTTTT | GCGTGGTTGGGGGAATGGTAG | 242 | 58 |
| *mox* | AAGGGTAAAGAAATGCGTCAAGA | CCCACAAACTCCATCAGTATCG | 177 | 58 |
| *zar1* | AGTCCAAACAACAGGTGACAAGTG | CTCCTTTTGGCATTTCCTACAGT | 212 | 58 |
| *zp3* | CAAAGGACTGGTTCCCACAAAT | GATGGGTATTCTGCTCCACACTA | 174 | 58 |
| *zp4* | GAATAACACTGAGCTGGAGGACAA | CTTTCTGATGCTTGACCATACGAT | 136 | 58 |
| *pnpla2* | ACGGATACTCCTGTTTGTTGTGA | TTTTGTAGATGTCTTCAGCCACG | 210 | 58 |
| *cidea* | AGAGGAGCGGGATTGCTAAAC | ATCTGAAGTCATAAGAGAGTGCGTA | 123 | 58 |
| *fabp4* | GAGTTTGACGAGACGACTGCTG | CTCCTTCTCGTAGGTCCTCACTG | 189 | 58 |
| *elovl6* | GTTTGACGAGAGAGGGGCGAT | CAACAATACTGAAGACAGCGAGG | 182 | 58 |
| *acsl5* | CGAACGGAGTCCTGAAGATTATT | GCTTCTTTGCCCAGTTTAGTCAT | 298 | 58 |
| *cox5b* | ACTTCTCCTCCGAACCTGTAGCA | CATCTGTCGGGATTCCGTTCAT | 175 | 58 |
| *cox6a* | GTTGGAGGAACACAAAGACCTACA | CTCTGATTGACGAAATGTGGTGTAA | 183 | 58 |
| *capn2* | TTGACCCTTGAGTGAGTTCTTACAG | CTCAATACGAGAGTAGTGACGCAAG | 151 | 58 |
| *elapor2* | TCATCTGTCAATCAACCATCATCC | TTGCCTGGGTTGACCTGTAGAA | 204 | 58 |
| *tp53bp2* | CAACACTGTGAACTCTGGGTCTG | TCTTCTGGTAGAGCAACTTCTGGAT | 296 | 58 |
| *tp53i3* | GACTATCCCCAGACCTCAACCC | GAGCAAAGCCATTACCCTGTCAT | 217 | 58 |
| *boka* | TGTGGTTGGGTGACGAGTTGGA | GCTCTCTGATGCTACTGTGATGTTG | 89 | 58 |
| *bnipl* | CACTTGAAGACGACGATGACACG | TCATCTTCCAAGTCTGGGTCGTAG | 112 | 58 |
| *perp* | ATCTTCCAGGTCATCGCTCTAAT | AGCCGTAAGCCCAGGTGTAGTAA | 91 | 58 |
| *bad* | GAACAAACGCAGCAGGTTACTCA | TTCCTCATCTCCCCTTTGTCTAAC | 290 | 58 |
| *caspase8* | ATCCAAAAAGACTCTCAAAAAGCG | TATCTCTATCTCAAAGCCCAGCCA | 228 | 58 |
